# Low budget analysis of Direct-To-Consumer genomic testing familial data

**DOI:** 10.12688/f1000research.1-3.v1

**Published:** 2012-07-16

**Authors:** Gustavo Glusman, Mike Cariaso, Rafael Jimenez, Daniel Swan, Bastian Greshake, Jong Bhak, Darren W Logan, Manuel Corpas

**Affiliations:** 1 Institute for Systems Biology, 401 Terry Avenue North, Seattle, WA 98109-5234, USA; 2 River Road Bio LLC, Potomac, MD 20854-3976, USA; 3 European Bioinformatics Institute, Wellcome Trust Genome Campus, Hinxton, Cambridge, CB10 1SD, UK; 4 Oxford Gene Technology, Begbroke Science Park, Begbroke, Oxfordshire, OX5 1PF, UK; 5 Molecular Ecology Group, Biodiversity and Climate Research Centre, Frankfurt am Main, Senckenberganlage 25, D-60325, Germany; 6 Theragen BiO Institute, TheragenEtex Inc, AICT building, Lui-dong, Youngtong-gu, Suwon 443-370, Korea, South; 7 Wellcome Trust Sanger Institute, Wellcome Trust Genome Campus, Hinxton, Cambridge, CB10 1SA, UK; 8 The Genome Analysis Centre, Norwich Research Park, Norwich, NR4 7UH, UK

## Abstract

Direct-to-consumer (DTC) genetic testing is a recent commercial endeavor that allows the general public to access personal genomic data. The growing availability of personal genomic data has in turn stimulated the development of non-commercial tools for DTC data analysis. Despite this new wealth of public resources, no systematic research has been carried out to assess these tools for interpretation of DTC data. Here, we provide an initial analysis benchmark in the context of a whole family, using single nucleotide polymorphism (SNP) data. Five blood-related DTC SNP chip data tests were analyzed in conjunction with one whole exome sequence. We report findings related to genomic similarity between individuals, genetic risks and an overall assessment of data quality; thus providing an evaluation of the current potential of public domain analysis tools for personal genomics. We envisage that as the use of personal genome tests spreads to the general population, publicly available tools will have a more prominent role in the interpretation of genomic data in the context of health risks and ancestry.

## Introduction

Direct-to-Consumer (DTC) genetic testing is a relatively new commercial endeavor offering access to personal genomic tests to the general public. Individuals wishing to learn about their genomes today enjoy a range of options. DTC providers typically offer chip-based genotyping of genome-wide markers, currently in the range of hundreds of thousands to a million single-nucleotide polymorphisms (SNPs). This current wealth of personal genomic data is likely to grow at an increasing pace as DNA sequencing become ubiquitous in personal genome testing. Genome sequencing allows elucidation of not just SNPs, but copy number variants (CNVs), insertions, inversions and many other genomic features currently underrepresented in personal genome analyses. Yet personal SNP data has proven to be a valuable resource for making personalized inferences about the risk of developing medical conditions, the probability of having certain phenotypic traits, and one’s likely ancestral origins ^1, 2^. Taking advantage of the growing body of statistical associations accumulated in the scientific literature, DTC providers have been able to offer personalized genomic ‘reports’ that present accessible scientific information of relevance to their customers’ observed genotypes. It is precisely in these genomic annotations where customers realize the value of their DTC product purchase.

A feature of DTC genomic test interpretation is that, being a commercial product, genomic annotations and analysis tools are proprietary and not freely available to the research community. This has motivated the parallel development of public resources and low cost genotype analysis tools. SNPedia ^3^ is a wiki-styled resource that collects and annotates SNPs from the scientific literature and provides tools with which to associate these annotations to those observed in DTC genomic tests. openSNP ^4^ is a public resource that collects genotypes from people willing to share them, allows annotation of phenotypes, and the search of occurrences of a particular SNP in scientific publications using Mendeley ^5^. Although all resources, public or commercial, are limited by the reliability of the data available for any given marker, public and low cost resources have the potential of engaging community wide efforts (‘crowd-sourcing’) to an extent to which closed commercial applications cannot.

We decided to explore the extent to which phenotype inference and genotype analysis can be carried out solely using existing public or very low cost resources. This is motivated by our belief that no DTC company will ultimately be able to match the rapid pace of genomic data accumulation and annotation that the research community is producing. Apart from SNPedia or openSNP, a wiki-based model, perhaps integrated with existing genetics resources such as the Gene Wiki ^6^ or Gene Wiki+ ^7^, may offer a good solution for rapid, accurate and comprehensive community annotation of personal genomic data.

In this paper we carry out a systematic analysis of DTC genomic data from a family of five blood relatives using mostly public annotations and tools. We present a) our findings related to the quality of the data, b) a comparison of the similarity between members of the family and an undisclosed individual of a different ethnic background and c) phenotype inferences as described by SNPedia trait annotations. We also incorporate analysis of DTC exome sequence data to supplement the genotype findings of one individual. We thus offer a pioneering methodical study of a whole family analyzed using only DTC data. Since comparable data could, in principle, be bought by any individual, this study benchmarks the personal genomic analyses available to non-experts using open, web-based tools.

## Results

**Figure 1.**
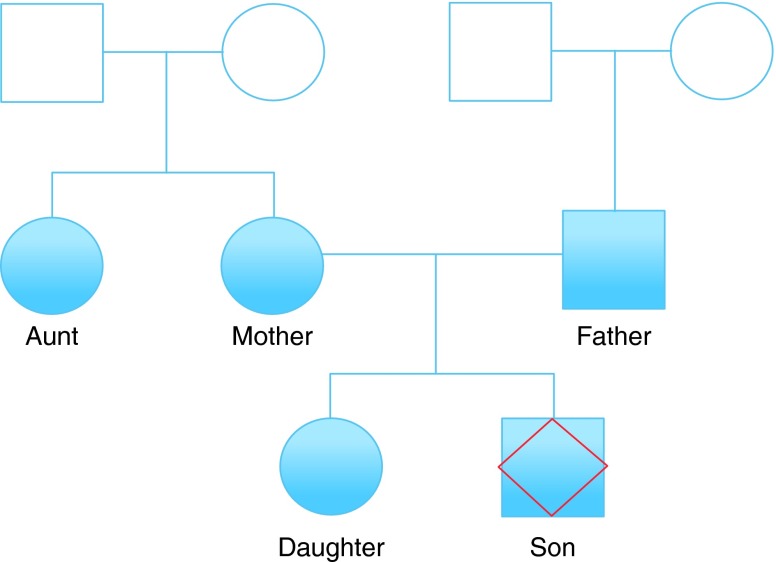
Family tree analyzed using DTC genotyping services. Squares and circles denote male and female respectively. Filled shapes represent those for which genome data is available. ‘Son’, the individual whose exome was sequenced, is denoted with a red diamond. Other family members include Father, Mother, Daughter and Aunt, who is Mother’s sister.

We analyzed DTC genomic data from a family of five of self-reported European ancestry, two males and three females across two generations ( Figure 1).

**Figure 2.**
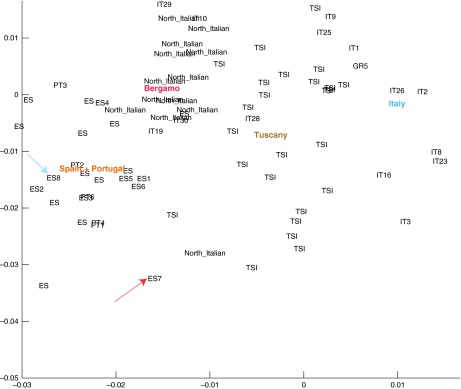
Admixture analysis of individuals from Southern Europe from the Eurogenes Genetic Ancestry Project. Mother (ES7) is denoted by a red arrow and Father (ES8) by a blue arrow. Mother and Father are the only family individuals included here as they have the most divergent genotypes within the family.

The family has lived in the southern-most region of Western Europe (Andalusia, Spain) for at least 4 generations. Principal component analyses of ancestry informative markers confirm tight parental clustering with Southern European populations ( Figure 2). We thus expect their ethnic background to be relatively homogeneous. The CEU HapMap ethnic group ^8^ was taken as the reference genotype for SNPedia phenotype predictions. Two kinds of SNP chip were used in this analysis, the 23andMe ^9^ versions 2 and 3. All family members except ‘Son’ (denoted with red diamond in Figure 1) were tested with version 3. Son was tested using version 2 and whole exome sequencing.

### Family SNP chip analysis

Whole family genotypes provide an additional genetic context that individual data analyses cannot offer, enabling enhanced error correction and inheritance state analysis ^10^. We found that data downloaded from 23andMe at different times may vary, probably as a consequence of changes in genotype-calling algorithms. To ensure consistency in our analyses, we downloaded the most recent data, for all family members, on May 30 ^th^ 2012.

23andMe SNP chip genotype data 23andMe genotype data for Mother, Father, Son, Daughter and Aunt. Son is 23andMe version 2 data and the rest of the family are 23andMe version 3 data.

**Figure 3.**
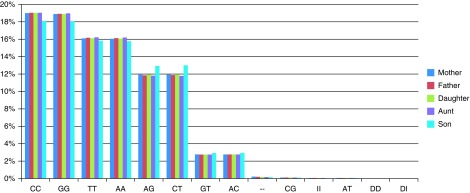
A distribution of all the different occurring genotypes as a percentage of the total for all individuals is shown. For the purposes of unbiased comparison, only autosome data is included. I and D indicate insertion and deletion, respectively. Son’s percentages (v2) show slight differences to all other v3 individuals whose genotype proportions are more similar.

In order to facilitate comparison between different genotypes, we excluded non-autosomal SNP data (chromosomes X, Y and MT). All v3 chips had a total of 930,342 autosomal SNPs; the v2 SNP chip had 556,694. The reported ‘no call’ rate (shown as ‘- -’ in the downloaded data) varied slightly for each individual ( Table 1), but overall, when genotypes are expressed as percentages of the total, some differences are observed for Son (v2) when compared to all other v3 individuals ( Figure 3). V3 individuals show a very similar distribution of genotypes.

**Table 1.** Summary of reported no-call rates for all 23andMe chips included in this study.

Family member	No call rate	Chip version
Mother	0.24%	v3
Father	0.21%	v3
Daughter	0.19%	v3
Aunt	0.17%	v3
Son	0.16%	v2

### Calculation of error rates

23andMe reports a 98% or greater call rate ^11^, meaning that the chip can provide accurate data for more than 98% of those variants in any particular person. When an allele variant present in heterozygous state is "undercalled" (not observed), the locus may be reported as being homozygous for the other variant, leading to missed heterozygosity. Such sites may significantly impact the disease risks predicted for the individual. Under the simplifying assumption of uniform undercall probability, we estimated the number of heterozygous sites mistakenly reported as homozygous ( Table 2). This means that for Son, 1 in every 400 sites is mistakenly called. For Father, 1 in every 200. Next, we analyzed Mendelian Inheritance Errors (MIEs). If at one site a reported genotype is ‘CC’ but the genotypes for both parents is ‘TT’, one possible explanation is that one of the parents is actually heterozygous ‘CT’ but was undercalled as ‘CC’, and likewise the son is heterozygous ‘CT’ but was undercalled ‘TT’. Given 5 people, there are 10 possible pairwise relations. Four of these represent direct parent/offspring relations, for which discrepancies can be counted as MIEs ( Table 3).

**Table 2.** Number of heterozygous sites mistakenly reported as homozygous (based on the undercall rate, in autosomes).

Member	Undercall	Heterozygous to Homozygous
Son	0.25%	661
Daughter	0.53%	2007
Mother	0.60%	2269
Father	0.50%	2010
Aunt	0.51%	1905

**Table 3.** Mendelian Inheritance errors as estimated by direct parent/offspring relations.

Relation	MIEs
Son/Father:	36
Son/Mother:	24
Daughter/Father:	108
Daughter/Mother:	129

One SNP in the Daughter (chr4:7, 9957, 622) is in disagreement with both parents: Father=CC, Mother=CC, Daughter=TT.

For the remaining 6 relationships, only part of the genome is expected to be identical by descent (IBD). Fully incompatible sites can be numbered as "pseudo-MIEs" ( Table 4).

**Table 4.** Incompatible sites between remaining relationships, identified as pseudo-MIEs.

Relation	Pseudo-MIEs
Son/Daughter [siblings]:	7,777
Mother/Aunt [siblings]:	15,401
Son/Aunt:	17,390
Daughter/Aunt:	30,215
Father/Mother [unrelated]:	53,937
Father/Aunt [unrelated]:	54,522

The reduced numbers of MIEs and pseudo-MIEs between the Son and all other family members were due to the lower total number of SNPs assayed for the Son. The Daughter has more MIEs relative to the Mother than relative to the Father. This may be due to having inherited a few deleted segments leading to a hemizygous state: hemizygous sites are reported as homozygous for the allele present, leading to an accumulation of apparent MIE sites. For example, at chr2:41093584 (rs12465519) ( Table 5). The ISCA analysis explains how we inferred a deletion from observed discordant genotypes.

**Table 5.** Example of hemizygous site reported as homozygous.

Status	Father	Mother	Daughter
Reported	GT	GG	TT
Actual	GT	G-	T-

A deletion inferred from mismatching genotype data The genotypes observed for the Mother (M) and Daughter (D) in the range 41,092,148-41,101,972 of chromosome 2 are mutually incompatible (Mendelian Inheritance Errors, highlighted in red). Genotypes for the Father (F) are shown for reference. Heterozygous sites are highlighted in grey. The simplest explanation for the cluster of incompatible genotypes is the presence of a deletion in the Mother''s genome, inherited by the Daughter. Both Mother and Daughter are thus hemizygous in this region. Right panel: inferred genotypes, showing the deleted segment inherited from Mother (blue) and the phased haplotype inherited from Father (yellow).

### SNP similarity analyses

We compared the 23andMe genotypes of the family members at three levels: 1) pairwise all-against-all comparison, 2) inheritance state analysis in family quartets, and 3) analysis of population admixture.

*1. Pairwise comparison.* We performed an all-against-all genotype comparison among all family members. As an external point of reference, we included in our analysis a male individual of Indian ethnic background (denoted as *non-CEU*, chip version 3). Table 6 shows a summary of the SNP similarity patterns found among all family members and the non-CEU individual. When comparing the similarity of tested SNPs between family members (for information on how similarity scores are calculated see Methods section) we find that Son is most similar to Daughter (85.7%), then to Father (83.8%) and Mother (83.7%), and least to Aunt (78.7%) and the non-CEU individual (75.4%). Among individuals sharing the same platform, we find that siblings, Aunt and Mother share the greatest number of identical genotypes (84.5%). When comparing whose SNPs are the most similar to Father, neither Mother nor Aunt are found significantly different (p-value = 0.5561). The non-CEU individual has, on average, a similarity of 75.3% to all family members tested on platform v3. Contrary to our expectation that both Sister and Son should be equally similar to Mother and Father, we found that Daughter is 84.8% identical to Mother and 85.0% to Father, potentially explained by the inheritance of a deletion (see previous section). This difference Father/Daughter vs. Mather/Daughter similarity was found significant (p-value = 1.731e-05). Son however, exhibited 83.7% identity to Mother and 83.8% to Father and his tested SNPs were not found to be significantly different to either of them (p-value = 0.2751).

**Table 6.** Similarity comparison of all-against-all family genotypes plus a non-European (non-CEU) male of Indian ethnic background. Matches denote identical genotypes for the same SNP (e.g. AA/AA); half-match, only one of the alleles is identical (e.g. AT/AA) and conflict means both alleles are different (e.g. CG/AT).

Mother	matches: half matches: conflict:	930342 0 0	100.0% 0.0% 0.0%
	identity: difference:	930342 0	100.0% 0.0%
	total:	930342	100.0%
Father	matches: half matches: conflict:	537331 335157 57854	57.8% 36.0% 6.2%	930342 0 0	100.0% 0.0% 0.0%
	identity: difference:	704910 225433	75.8% 24.2%	930342 0	100.0% 0.0%
	total:	930342	100.0%	930342	100.0%
Daughter	matches: half matches: conflict:	650399 276334 3609	69.9% 29.7% 0.4%	653926 273483 2933	70.3% 29.4% 0.3%	930342 0 0	100.0% 0.0% 0.0%
	identity: difference:	788566 141776	84.8% 15.2%	790668 139675	85.0% 15.0%	930342 0	100.0% 0.0%
	total:	930342	100.0%	930342	100.0%	930342	100.0%
Aunt	matches: half matches: conflict:	661316 250278 18748	71.1% 26.9% 2.0%	537723 335063 57556	57.8% 36.0% 6.2%	586549 310933 32860	63.0% 33.4% 3.5%	930342 0 0	100.0% 0.0% 0.0%
	identity: difference:	786455 143887	84.5% 15.5%	705254.5 225088	75.8% 24.2%	742016 188327	79.7% 20.2%	930342 0	100.0% 0.0%
	total:	930342	100.0%	930342	100.0%	930342	100.0%	930342	100.0%
Son	matches: half matches: conflict:	357419 168682 1807	67.7% 32.0% 0.3%	358003 168344 1561	67.8% 31.9% 0.3%	386014 132693 9201	73.1% 25.1% 1.7%	321410 187749 18749	60.9% 35.6% 3.6%	556694 0 0	100.0% 0.0% 0.0%
	identity: difference:	441760 86148	83.7% 16.3%	442175 85733	83.8% 16.2%	452361 75548	85.7% 14.3%	415285 112624	78.7% 21.3%	556694 0	100.0% 0.0%
	total:	527908	100.0%	527908	100.0%	527908	100.0%	527908	100.0%	556694	100.0%
Non-CEU	matches: half matches: conflict:	530234 340807 59106	57.0% 36.6% 6.4%	528933 341639 59575	56.9% 36.7% 6.4%	528747 342833 58567	56.8% 36.9% 6.3%	530171 341419 58557	57.0% 36.7% 6.3%	283484 209091 36191	53.6% 39.5% 6.8%	934670 0 0	100.0% 0.0% 0.0%
	identity: difference:	700638 229510	75.3% 24.7%	699753 230395	75.2% 24.8%	700164 229984	75.3% 24.7%	700881 229267	75.4% 24.6%	388030 140737	73.4% 26.6%	934670 0	100.0% 0.0%
	total:	930147	100.0%	930147	100.0%	930147	100.0%	930147	100.0%	528766	100.0%	934670	100.0%
		Mother		Father		Daughter		Aunt		Son		Non-CEU	

*2. Inheritance state analysis.* The availability of nuclear families with two or more offspring enables the identification of inheritance states ^10^. These represent whether the offspring inherited the same alleles from both parents ("identical" state), the same allele from one parent but different alleles from the other ("haploidentical" state, maternal or paternal according to the parent from which the same allele was inherited), or different alleles from both parents ("nonidentical" state). Some family genotypes are consistent with all inheritance states (e.g. when all family members are homozygous) and are thus non-informative. Some family genotypes are consistent only with a subset of the inheritance states. For example if both parents are heterozygous A/G, and both offspring are homozygous A/A, clearly the offspring inherited the same alleles from both parents, which is consistent only with the "identical" state. Some family genotypes are consistent with two inheritance states. By combining the evidence from individual SNPs along a chromosome, it is possible to identify contiguous blocks of consistent inheritance, bounded by recombination events in either parent ( Figure 3). The overall fraction of the genome present in each inheritance state ( Figure 3 inset) deviates little from the expected 25%.

We found that 23andMe genotypes are sufficient for performing this analysis and identifying well-defined inheritance state blocks, despite covering a very small fraction of the genome (approx. half a million shared SNPs, limited by the lower density version used for Son). This is due to the largely uniform sampling of SNPs along the genome.

*3. Admixture analysis.* Visualization of admixture for Mother and Father was done with ADMIXTURE ^12^ in the context of other similar Southern European individuals. Figure 2 showed an admixture mapping for a selection of individuals from the Eurogenes Genetic Ancestry project ^13^. Mother was denoted as ES7 (red arrow) and Father as ES8 (blue arrow). Mother and Father seemed to be markedly different yet in and around the Portuguese and Spanish cluster of individuals.

### Combining SNP chip data and exome SNPs in Son for SNPedia annotation

To leverage all genotype data contained in all different sources, we combined the SNP chip data v2 (574,406 SNPs) with those found in Son’s exome data. 10,203 genotypes were annotated in SNPedia when exome SNPs were pooled with the SNP chip version 2 (generated on 11 ^th^ June, 2012). Processing just the exome, only 925 genotypes were found annotated in SNPedia. It is not surprising that so few additional SNPs were found, as the exome comprises a very small percentage of the total genome. Two SNPs were discovered to have conflicting genotypes between the two platforms: *rs12344615* reported as ‘AG’ and ‘GG’ and *rs2290272* reported as ‘CT’ and ‘TT’ respectively. The most likely informative SNPs from the exome data are summarized as judged by their observed frequency in HapMap ^8^.

SNPs from the Son’s exome data Summary of the most likely informative SNPs from the Son’s exome data as judged by their observed frequency in HapMap.

### Exome data summary statistics

For the analysis of similarities between genotypes only 23andMe data was analyzed. Although exome sequencing is not widely marketed yet within the DTC providers as an option, this is likely to change in the near future. With our current budget constraints, we were able to sequence Son’s exome and relevant SNP and variation data was added for further analysis. A BAM file was created out of 4 FASTQ files downloaded from a server from the Beijing Genomics Institute. A compressed VCF file was also created. A total of 2.54 Gigabases of sequence was aligned at high quality. Summary metrics of the exome were calculated using Picard ^14^ and showed a minimum of 61.42% of the on-target regions were covered with a depth at least 20x. Genotyping with GATK ^15^ identified 37,702 variations relative to the reference genome (GRCh37). This was noted to be lower than expected if additional samples had been genotyped concurrently. 97% of the variants identified were within a known gene, 58% of them overlapped a protein domain and 5,565 were non-synonymous (15%) with serious predicted consequences on the protein product (as determined by SIFT ^16^, PolyPhen ^17^ or Condel ^18^). Of these 5,565 potentially pathogenic SNPs, 413 had not been previously identified (verified against dbSNP release 132). This represents a normal figure for the private, novel, non-synonymous changes carried by most individuals. No more serious, novel changes were identified (such as stop codons gained or lost).

### Visualization with SNPedia tools

We used SNPedia’s Hilbert curve visualization tool to compare chromosomes between different individuals. Figure 5 shows an example of a Hilbert curve comparison between chromosome 1 of Mother and all family members and the non-CEU individual. Each pixel corresponds to a SNP, colored according to four categories: match (light blue), half-match (dark blue), mismatch (red) and no data (grey). Two patches of light blue are apparent in the Mother-Aunt comparison of chromosome 1, and more appear in other chromosomes (not shown). These correspond to ‘identical’ segments in which the Mother and the Aunt inherited identical haplotypes from both their parents.

ISCA analysis for the quartet (missing grandfather), (missing grandmother), mother and aunt This figure shows that the haploidentical states are well distinguished (contiguous green or yellow segments). Lacking information on the grandparents, it is impossible to distinguish between haploidentical maternal and haploidentical paternal for the mother-aunt comparison, hence the haploidentical states are shown as mixtures of green and yellow.

**Figure 4.**
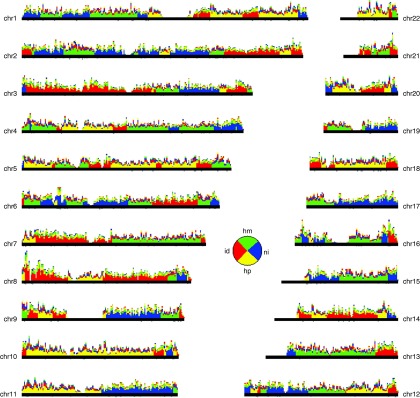
Inheritance State Consistency Analysis (ISCA) plot for the Father-Mother-Son-Daughter quartet, depicting for each autosome the number of informative SNPs supporting each of the four possible inheritance states: "identical" ("id", red), "haploidentical maternal" ("hm", green), "haploidentical paternal" ("hp", yellow) and "nonidentical" ("ni", blue). SNPs consistent with two inheritance states contribute 0.5 weight to each. SNP counts are binned in non-overlapping 1 Mb windows; within each window, the four inheritance states are sorted by decreasing level of support. Regions without support typically overlap centromeric repeats and heterochromatic regions. Pie chart inset: fraction of the genome observed in each inheritance state.

**Figure 5.**
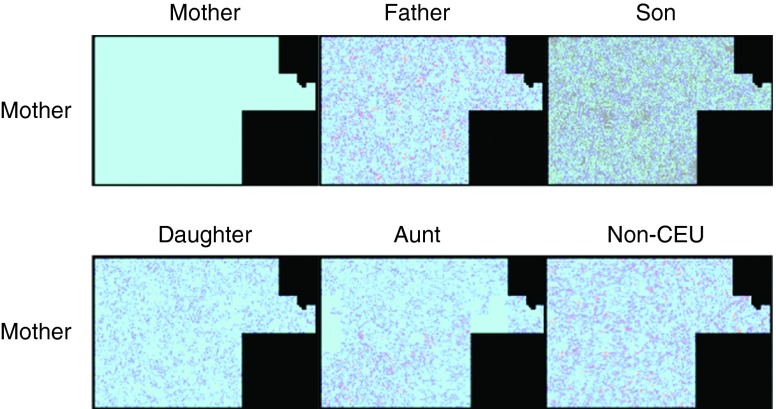
A graphical representation of the SNPs from chromosome 1 for Mother com pared with herself, Father, Son, Sister, Aunt and non-CEU. Each pixel represents a SNP. Light blue represents match, dark blue half-match and red conflict. SNPs in Son that are not present in the genotypes of the other individuals are represented in grey.

The next most similar graph can be seen to be the Mother/Daughter comparison. Much of the graph comparing Mother and Son is grey, representing SNPs present only on 23andMe v3 but absent from v2.

### Inference of phenotypes using SNP data

23andMe SNPs for which SNPedia annotations are available Each file contains all SNPs in the individual matching an annotated SNP in SNPedia. SNPedia annotations contain a magnitude value (subjective measure of the importance of the potential phenotypical effect) and a phenotype description of the condition of particular genotype affects.

We inferred phenotypes from the observed genotypes, by comparing to all available SNPedia SNP annotations. We analyzed family genotypes using the Promethease tool ^19^, which allows annotation of observed genotypes from DTC analyses with SNPedia-annotated phenotype associations. These are collected directly from the scientific literature. All family members were analyzed using Promethease and associated phenotypes were collected for further interpretation. SNPedia’s annotation *Magnitude*, denoting a subjective degree of importance for an observed trait as judged by the curator, was used to discriminate between traits that should be considered further in our analyses. A magnitude of 0 denotes a common genotype for which no associated phenotypic data is known. Magnitude >3 is defined as ‘probably’ interesting. The maximum number is 10. In order to compare family phenotype annotations, all SNPs or ‘genosets’ (groups of SNPs) with equal or greater than magnitude 3 were extracted and summarized in Table 5. Results for Son are not directly comparable as he has fewer SNPs analyzed.

**Table 7.** A comparison of all SNPedia annotations with Magnitude >= 3 for all family members. Traits have been classified according to the general condition they relate. Red boxes are indicative of a particular phenotype being predicted in the individual. Descriptions for every matched phenotype, extracted directly from SNPedia, are shown in the right column.

Condition	Mother	Father	Daughter	Aunt	Son	Phenotype
Baldness						7x risk of baldness according to an article in Nature. That site may require paid access; the abstract is accessible.
						2x increased risk of baldness 2x increased risk of baldness
Diabetes						Increased risk for type-2 diabetes
						1.3x increased risk for type-2 diabetes
Cardiovascular/Thrombosis						1.7x increased risk for heart disease. People with this genotype and a long history of high blood sugar are at 7x risk of CAD
						1.5x increased risk for CAD; 1.5x higher risk for coronary artery disease
						7.3x increased risk of hypertension
						Watch out for high fat in diet
						2.6 times higher odds of developing early stent thrombosis
Cancer						Increased risk of various types of cancer. This variant increases risk of numerous types of cancer in many studies. It is in a microRNA
						2–3x higher prostate cancer risk if routinely exposed to the pesticide fonofos
Metabolism						You have 2 variations in MTHFR which influence homocystine levels. People with gs193 are more strongly affected.
						Impaired NSAID drug metabolism, which is a risk factor for gastrointestinal bleeding when taking any of these medications: aceclofenac, celecoxib, diclofenac, ibuprofen, indomethazine, lornoxicam, meloxicam, naproxen, piroxicam, tenoxicam and valdecoxib. You have one of these *CYP2C8*3 (rs11572080 and rs10509681) *CYP2C9*2 (rs1799853) *CYP2C9*3 (rs1057910)
						CYP2C19 Intermediate Metabolizer. Your body breaks down some medicines at a slightly slower than normal rate (which is represented by gs150). Individuals with gs152 genotypes have even slower metabolism. *anti-epileptics (such as diazepam, phenytoin, and phenobarbitone) *anti-depressants (such as amitriptyline and clomipramine) *anti-platelet drug clopidogrel (Plavix) *anti-ulcer proton pump inhibitors like omeprazole (trade names Losec and Prilosec), esomeprazole (trade name Nexium), and lansoprazole (Prevacid) *hormones (estrogen, progesterone).
						Higher odds of alcoholic liver disease, increased liver fat alcohol seems to be 3x more damaging to your liver than typical. Higher risk for developing fatty liver, fibrosis, and fibrosis progression, with a per allele odds ratio of 2.55, 3.13 and 2.64, respectively. news
Warfarin Metabolism						Approximately 30% of people are intermediate metabolizers of the popular anticoagulant Warfarin and would probably need a decreased dosage. This due to rs1799853 or rs1057910 respectively leading to the CYP2C9*2 or CYP2C9*3 alleles. For prodrugs that require activation by CYP2C9, an alternative treatment or increased dose should be considered. See also gs126
						Probably impaired Warfarin metabolism.
						Approximately 7–10% of people are poor metabolizers of the popular anticoagulant Warfarin and would probably need a decreased dosage. This due to mutations in rs1799853 or rs1057910 causing an inactive CYP2C9 gene. You are at increased risk of drug-induced side effects due to diminished drug elimination. Prodrugs dependent on CYP2C9 metabolism may fail to generate the active form of the drug.
Miscellaneous						Substantially increased odds of developing V617F-positive MPN.
						You are heterozygous at all 3 of the SNPs which are known to influence the ability to taste bitterness. This means you are better than average at detecting bitter tastes while young, but that this ability will decrease to less than average during adulthood. As a child you will probably hate brussel sprouts, and by early adulthood will discover that olives and brussel sprouts now taste good. A 2010 study shows the change bitter sensitivity which occurs over the lifespan (from bitter sensitive to less so) is more common in people with this genoset. Children with this genotype could perceive a bitter taste at lower PROP concentrations than could heterozygous adults. The threshold for adolescents was intermediate. The 3 SNPs are rs10246939, rs1726866, rs713598 in the gene TAS2R38.

Based on observed results, the maternal hereditary line seems to carry greater risks related to diabetes and cardiovascular/thrombosis related conditions. In addition, both males (Son and Father) have greater risk of baldness as well as mixed results in terms of their ability to metabolize drugs. All the family shares a common trait of substantially increased odds of developing V617F-positive Myeloproliferative neoplasms.

## Discussion

We have presented here, to our knowledge, the first systematic analysis of DTC genomic data using non-commercial or low cost resources, combining data from 5 blood-related family members. The purpose of this study does not lie in uncovering the phenotypic predictions or genetic findings in these individuals’ genomes. Instead we aimed to demonstrate to what extent, in principle, any individual can interpret their personal genome using only public resources with an affordable budget and no laboratory equipment. We stress that our goal is not to diminish the value of DTC industry services, which have catalyzed the access to, and interest in, personal genomics data in the wider public. However, as the adoption of DTC personal genomics tests becomes ever more widespread, we envisage community phenotype association and third party public tools to become more significant in the overall interpretation of personal genomics results.

Although we found the no-call rate to be comparable between both versions of the 23andMe platform, Mother had a greater undercall rate than the other individuals. Also, results given by no-call rates suggest that there might be some intrinsic differences between the two chips. Nevertheless the current number of samples analyzed is not large enough to make this conclusion, as only five chips of data analyzed do not provide any basis for their overall performance. Therefore our error estimation rates presented here should only be considered in the context of this analysis and not as representative of the DTC company’s overall quality scores.

Based on the no-call rate and the relative ratios of homozygous and heterozygous sites reported, we computed an ‘undercall’ rate and used it to estimate the number of heterozygous sites mistakenly reported as homozygous. The frequency of such events is small (~0.2%). Nevertheless, the fact that up to 2,000 heterozygous variants may be missed is a reminder that interpretation of personal disease risks should be done with caution. Findings of potential medical relevance should always be verified for correctness. Wherever possible, it is beneficial to perform the analysis in the context of families: identification of MIEs and State Consistency Errors ^10^ is a powerful tool to assess genotyping quality.

The similar level at which identical genotypes is shared between the relatives and the non-CEU individual is consistent with the ethnically close background for the family members. This, however, does not suggest that Mother or Father are directly related. In fact, when performing an admixture analysis with unrelated individuals of Southern European descent ( Figure 2) it is clear that while the parents cluster within reasonably close distance to other Spanish individuals, they display a typical level of genotype sharing between two people from the Iberian Peninsula.

We also indicated that when comparing genotype similarities between siblings and parents, we found that Daughter was significantly closer to Father than Son was. Although the expectation was that both Daughter and Son should be equally similar to both parents, these unexpected results may be the reflection of bias in the subset of markers used in the DTC analysis. These results are not therefore indicative of Daughter’s genome being closer to either parent, as most of the genome is missing and hence any inference in this respect cannot be made. In the context of SNP analysis, however, it is worthwhile reporting such SNP differences as these will influence the overall results reported back to the DTC customer. This in turn may explain why observed susceptibility risks vary among family members when comparing their phenotypic annotations.

The identification of blocks of identical, haploidentical or nonidentical genotype between family members (e.g. Son and Daughter in Figure 4), highlights the location of meiotic recombinations. These blocks provide well defined expectations for whether the genes included in them should display similar or distinct genotypes. This information is valuable in the context of genetic research ^10^ but also to the general public, to predict shared phenotypes among family members. Within ‘identical’ blocks, siblings are essentially identical twins: this fact gains special personal meaning in the context of DTC genetic analysis.

Publicly curated data, like that available in SNPedia, is exposed to error due to human mistakes or malicious intent. Although this is a legitimate concern, it has been shown that with similar public annotation resources (Wikipedia for example), vandalism rates are very low in areas of specialist academic interest ^20^. As these open access resources mature and grow, maintaining accuracy will be an important consideration for the contributing community.

In this study we have not found any annotated genotype that is likely to raise significant health concerns among the family individuals. It is inevitable, however, that as more individuals investigate their own personal genomes, and more statistical associations are uncovered, genotype/phenotype correlations with serious health implications will be accessible through open access resources. Interpreting such information appropriately is bound to be difficult for individuals who are not expert geneticists; this poses special ethical challenges from the point of view of how to present the data. DTC companies have recognized this ethical issue by implementing additional access controls to some particularly sensitive annotations, such as those genotypes associated with a high risk of developing Alzheimer’s disease or some forms of breast cancer. To our knowledge, public annotation resources do not currently make such distinctions. We therefore urge caution when investigating personal genomic data for health risks, especially when using open access information. We recommend those who uncover personal genomic information of medical concern seek the advice of genetic counselors, who can interpret and advise on the context of what it really means to the tested individual.

## Methods

### SNP chip data

Five 23andMe genome analysis kits were purchased at two time points. Son’s kit was bought in May 2009 and was tested with 23andMe version 2 (~576,000 SNPs). The other 4 family members were analyzed in one batch, with kits bought in December 2010 and results returned in February 2011. The second batch used 23andMe’s chip version 3 with ~967,000 SNPs per genome analyzed. After discussion of results, consent was given by all family members of the family to publish their genotypes.

### Calculation of error rates

Assume N SNPs were tested, and of these, fN are truly heterozygous. We wished to compute f (heterozygous fraction) from the observed numbers of homozygous, heterozygous and failed SNPs.

For each SNP with a dbSNP ‘rs’ identifier, we assumed that 1) the SNP is biallelic, 2) it is present in diploid state. We further assumed a probability x of not observing a given allele (the "undercall rate"), and assumed this probability was equal for both alleles, at all sites. Finally, we assumed that the probability of observing a wrong allele was zero ("overcall rate").

If the true state of the SNP was heterozygous, the following could have happened. 1) If neither allele was observed (double undercall), the SNP was called "NULL" (with conditional probability = x ^2^). 2) If one allele was not observed (single undercall), the SNP was called "HOM" (conditional probability = 2x). 3) If both alleles were observed, the SNP was called "HET" (conditional probability = 1-2x-x2).

If the true state of the SNP was homozygous, there was only one type of allele to be observed. Thus, the following could have happened. 1) If the allele was not observed, the SNP was called "NULL" (conditional probability = x). Otherwise, 2) the SNP was called "HOM" (conditional probability = 1-x).

The expected frequencies for NULL, HET and HOM were:

- NULL’ = fx ^2^+(1-f)x
- HET’ = f(1-2x-x ^2^)
- HOM’ = (1-f)(1-x)+2fx = 1-x+f(3x-1)

Since HOM + HET + NULL = 1, the undercall rate x was given by solving:

x ^3^ + (1+HOM)x ^2^ + (3HET+2HOM-3)x + NULL = 0

The heterozygous fraction f was then given by: f = HET/(1-2x-x ^2^), and the number of "missing" heterozygous sites could be computed by: missing = (f-HET)N. Finally, the number of heterozygous sites reported as homozygous was given by:

het2hom = missing - N*NULL*HET/(HET+HOM)

### Extraction of SNPs from exome data

Raw reads were aligned to the reference GRCh37 using bwa 0.61 ^21^. Local realignment was performed around indels with the Genome Analysis Toolkit (GATK v1.4) ^15^ framework for variation discovery and genotyping using next-generation DNA sequencing data. Optical and PCR duplicates were marked in BAM files using Picard 1.62 ^14^. Original HiSeq base quality scores were recalibrated using GATK TableRecalibration and variants called with GATK UnifiedGenotyper. Indels and SNPs were hard-filtered according to Broad Institute best-practice guidelines ^22^ to eliminate false positive calls and produce the final VCF.

Son exome files The Fastq files represent the raw exome data for the son. The BAM files are derived from the fastq files by aligning the reads using a Burrows-Wheeler Aligner (BWA). The BAM file (.bam) is the binary version of a tab-delimited text file that contains sequence alignment data. The BAM file index (.bai) provides fast random access to the BAM file. The compressed VCF file (.vcf.gz) describes variant calls of the data in text format. Click here for additional data files.

### Phenotype inference using promethease

Promethease, a SNPedia tool for phenotype inference, was used for assignment of SNPedia annotations to observed SNPs. SNPedia annotations contain manually curated SNPs that summarize phenotype associations observed in a particular population. Phenotype associations were inferred using SNPedia’s SNP ids, which correspond to dbSNP ^23^. In our analysis only SNPedia annotations of >= 3 Magnitude were examined. Magnitude is a subjective score that helps prioritize SNPs according to their expected importance and the phenotypic annotation itself in the form of free text. Magnitude is assigned by SNPedia entry curators.

### Calculation of similarity scores

We calculated similarity scores using our own Perl scripts and MySQL. Similarity between any given two individuals was calculated as the total number of matching SNPs plus half-matches divided by 2. Negative results were counted as the total number of conflicts plus the number of half-matches divided by 2. As the platforms used are different for Son as compared to the rest, Son can only be compared relative to himself and not against other individuals. Similarities between individuals were statistically tested using the R package for Pearson’s Chi-squared with Yates’ correction.

## Consent

Written consent for publication of their genotype and phenotype was obtained from all family individuals.
