# Supplementary figures and images for: Low budget analysis of Direct-To-Consumer genomic testing familial data
Source: F1000Res. 2012 Jul 16;1:3. [Version 1] doi: 10.12688/f1000research.1-3.v1 (PMC3941016; doi:10.12688/f1000research.1-3.v1)

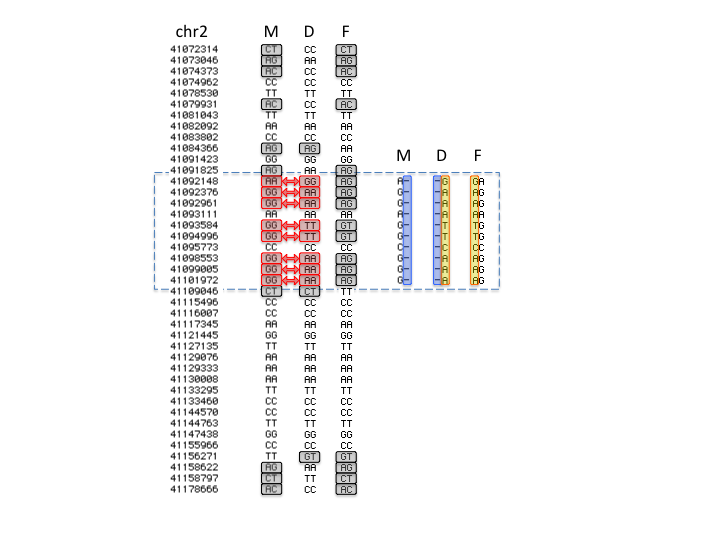

Supplement: A deletion inferred from mismatching genotype data — The genotypes observed for the Mother (M) and Daughter (D) in the range 41,092,148-41,101,972 of chromosome 2 are mutually incompatible (Mendelian Inheritance Errors, highlighted in red). Genotypes for the Father (F) are shown for reference. Heterozygous sites are highlighted in grey. The simplest explanation for the cluster of incompatible genotypes is the presence of a deletion in the Mother''s genome, inherited by the Daughter. Both Mother and Daughter are thus hemizygous in this region. Right panel: inferred genotypes, showing the deleted segment inherited from Mother (blue) and the phased haplotype inherited from Father (yellow). [file f1000research-1-103-s0005.tgz › supplFig2.png]

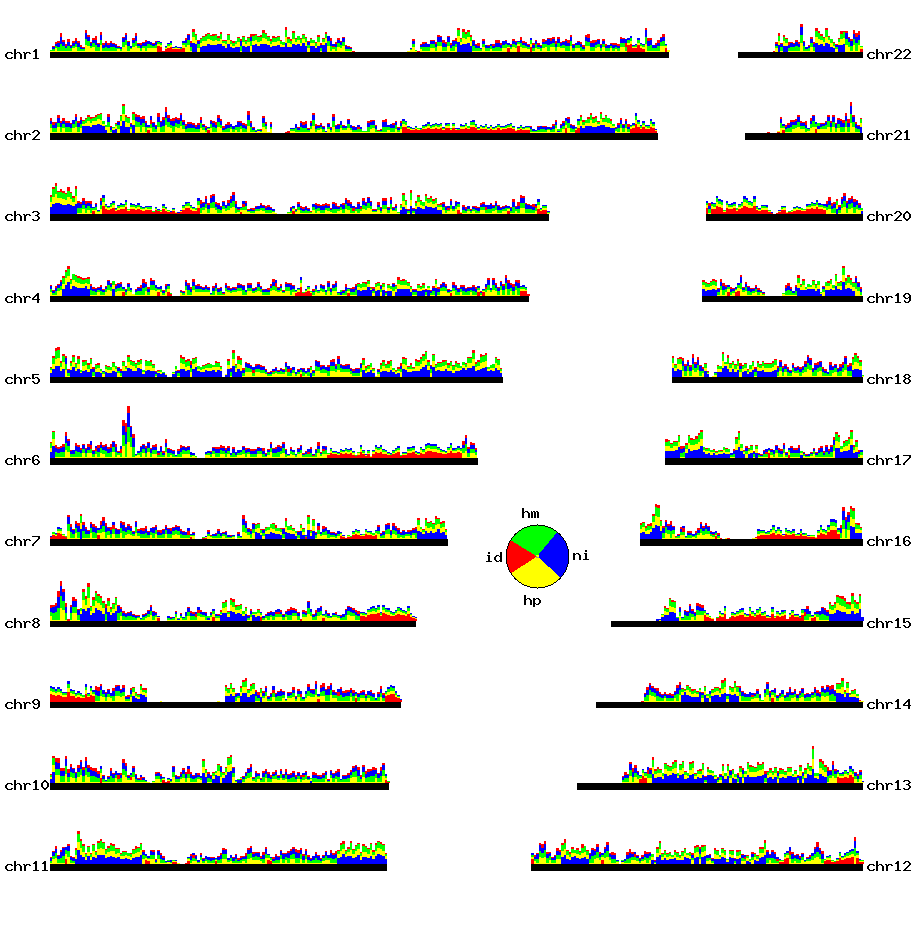

Supplement: ISCA analysis for the quartet (missing grandfather), (missing grandmother), mother and aunt — This figure shows that the haploidentical states are well distinguished (contiguous green or yellow segments). Lacking information on the grandparents, it is impossible to distinguish between haploidentical maternal and haploidentical paternal for the mother-aunt comparison, hence the haploidentical states are shown as mixtures of green and yellow. [file f1000research-1-103-s0003.tgz › supplFig1.png]
