# Supplementary material for: Low budget analysis of Direct-To-Consumer genomic testing familial data
Source: F1000Res. 2012 Jul 16;1:3. [Version 1] doi: 10.12688/f1000research.1-3.v1 (PMC3941016; doi:10.12688/f1000research.1-3.v1)
Supplement: Peer review history

# Peer review: Referee response for version 1

**Published:** 2012-07-26

**Authors:** Christian Gilissen

**Affiliations:** 1 Department of Human Genetics, Radboud University, Nijmegen Medical Center, Nijmegen, Netherlands

This is a well written and interesting article that describes analyses on genome-wide genotyping information from a DTC company.

**Major concerns**

- Although the analyses are carried out very well, the overall purpose and conclusion of the paper is rather mixed and somewhat unclear. The authors state that they “explore the extent to which phenotype inference and genotype analysis can be carried out solely using existing public or very low cost resources.” or as in the abstract “providing an evaluation of the current potential of public available analysis tools for personal genomics”.
- The discussion section focuses very much on the results of the different analyses, whereas that is (according to the purpose of the paper) not that relevant. Rather in that context it would be more interesting to speculate on other aspects (for example, how easy it is for a typical DTC customer to apply the publicly available tools, or how easy it is to (correctly) interpret the outcome of such tools) and to provide a real conclusion on “the extent to which phenotype inference and genotype analysis can be carried out solely using existing public or very low cost resources.”
- It is not clear whether the tools that were investigated compromise all public domain tools for these kinds of analyses. If the authors do not investigate all available tools, the authors should motivate their choice for the described tools

*Given the purpose of the paper, a table with the available tools and their “potential” would be very useful.*

**Minor concerns**

- The fact that “Son” was done with a V2 assay complicates all comparisons in the paper. It seems to paper would be a lot more straightforward if the authors used a V3 assay for the son as well.
- Page 7, “Exome data summary statistics”, authors mention that the numbers they find are what is to be expected. Please add a reference to substantiate this.
- Page 10, the cond. prob. of a heterozygous call for a heterozygous SNP reads “1-2x-x2” and should probably be “1-2x-x^2”. Similar with the expected frequency for NULL’.

Summary

- Is the title appropriate for the content of the article? **Yes**
- Is the abstract a suitable summary of the article? **Yes**
- Is the article well constructed and clear? **Yes, the article is well constructed, but could do with a conclusion and discussion that is more in line with the purpose of the paper. (see comments above)**
- Is there adequate analysis, including information on how the data were analyzed (e.g. programs, code, stats etc.)? **Yes**
- Are the conclusions sensible and balanced? **The conclusions does not answer the questions that were asked at the outset of the paper.**
- Have any potential biases or competing interests been disclosed? **Yes**

I have read this submission. I believe that I have an appropriate level of expertise to confirm that it is of an acceptable scientific standard.

# Peer review: Referee response for version 1

**Published:** 2012-07-23

**Authors:** Peter Robinson

**Affiliations:** 1 Institute for Medical Genetics,Universitätsklinikum Charité, Berlin, Germany

23andMe and several other companies have been offering Direct-to-consumer (DTC) genomic testing for several years now. In general, such tests seem to have a bad reputation amongst human geneticists because of the perception that individuals may not be able to interpret the findings in a useful way.

Additionally, although genetic testing has an important and widely accepted role in the diagnostics of Mendelian disease and several other areas, many feel that the clinical utility of genomic testing for common disorders such as heart disease or hypertension has not been shown to date. For instance, the Evaluation of Genomic Applications in Practice and Prevention Working Group (EWG) found insufficient evidence to recommend testing for the 9p21 genetic variant or 57 other variants in 28 genes to assess risk for cardiovascular disease in the general population (Genetics in Medicine (2010) 12, 839–843).

Be that as it may, has become well known and the range of variants now tested by companies such as 23andMe is increasing; with the advent of relatively cheap exome and even genome sequencing, it seems quite likely that DTC exome and genome sequencing will be offered in the not too distant future. Therefore, it is important to understand several aspects about DTC testing, including the quality of the raw product (i.e., are the genotype calls correct), the depth, correctness, and utility of annotations provided by DTC companies or widely available to the public, and perhaps most importantly, to know how typical consumers of DTC products use the findings for their own health care or, say, for planning life-style modifications.

This paper offers a detailed look at several of these aspects, and I think does an excellent job at providing the reader with a sense of what kind kind of data consumers of DTC products can expect. I think the paper would profit from a number of minor revisions. The paper essentially deals with two different topics. The first involves a number of computational quality control procedures that require a good deal of bioinformatics expertise (I doubt that much of this would be in the reach of most of the customers of DTC sequencing), ranging from a calculation of error rates, an analysis of Mendelian inheritance errors, genotype distributions, and admixture analysis. It was not entirely clear to me how the p-values reported for the SNP similarity analysis were calculated, and a more precise definition of how pseudo-MIEs were counted should be provided. The authors should provide full methodological details of how this analysis was performed. It is also something of a distraction that one of the samples was analyzed using the 23andMe v2 kit, while the rest of the family was analyzed using the v3 kit. I imagine simply that the Son was the person who “went first”, but the apparently different error characteristics of the two versions limit somewhat the findings of the paper.

The second major aspect of this paper involves analysis that could be done by non-specialists, using tools such as SNPedia. It was not entirely clear to me why the authors performed the analysis in Figure 5. The heading called “Inference of Phenotypes Using SNP data” is misleading because actually what the findings reveal is a genotype, say, a risk for going bald. However, if the son is not currently bald, then he does not now have the phenotype “baldness”. Instead, the only thing that can be inferred from the genotype is an increased risk of baldness. This is a very important distinction, and it is very important especially for the general public to realize that having a genotype that is associated with an increased risk of some phenotype does not necessarily mean that one will actually develop that phenotype.

It would be a nice addition to this paper to hear more about how the family reacted to these findings. Have the increased risks for some of the diseases mentioned led to life style changes? Did the family members report the findings to their physicians? Was a family member worried or uncomfortable about hearing the findings?

I have read this submission. I believe that I have an appropriate level of expertise to confirm that it is of an acceptable scientific standard.
